# Refining PD-1/PD-L1 assessment for biomarker-guided immunotherapy: A review

**DOI:** 10.17305/bb.2023.9265

**Published:** 2024-02-01

**Authors:** Marek Zdrenka, Adam Kowalewski, Navid Ahmadi, Rizwan Ullah Sadiqi, Łukasz Chmura, Jędrzej Borowczak, Mateusz Maniewski, Łukasz Szylberg

**Affiliations:** 1 Department of Tumor Pathology and Pathomorphology, Oncology Centre-Prof. Franciszek Łukaszczyk Memorial Hospital, Bydgoszcz, Poland; 2 Department of Cardiothoracic Surgery, Royal Papworth Hospital, Cambridge, UK; 3 Medical University Pleven, Pleven, Bulgaria; 4 Department of Pathomorphology, Jagiellonian University Medical College, Kraków, Poland; 5 Department of Obstetrics, Gynaecology and Oncology, Chair of Pathomorphology and Clinical Placentology, Collegium Medicum in Bydgoszcz, Nicolaus Copernicus University in Torun, Bydgoszcz, Poland

**Keywords:** Programmed cell death-1 (PD-1), programmed cell death ligand 1 (PD-L1), biomarkers, diagnosis, treatment response, digital pathology

## Abstract

Anti-programmed cell death ligand 1 (PD-L1) immunotherapy is increasingly crucial in cancer treatment. To date, the Federal Drug Administration has approved four PD-L1 immunohistochemistry (IHC) staining protocols, commercially available in the form of “kits,” facilitating testing for PD-L1 expression. These kits comprise four PD-L1 antibodies on two separate IHC platforms, each utilizing distinct, non-interchangeable scoring systems. Several factors, including tumor heterogeneity and the size of the tissue specimens assessed, can lead to PD-L1 status misclassification, potentially hindering the initiation of therapy. Therefore, the development of more accurate predictive biomarkers to distinguish between responders and non-responders prior to anti-PD-1/PD-L1 therapy warrants further research. Achieving this goal necessitates refining sampling criteria, enhancing current methods of PD-L1 detection, and deepening our understanding of the impact of additional biomarkers. In this article, we review potential solutions to improve the predictive accuracy of PD-L1 assessment in order to more precisely anticipate patients’ responses to anti-PD-1/PD-L1 therapy, monitor disease progression, and predict clinical outcomes.

## Introduction

In recent years, targeting programmed cell death-1/programmed cell death ligand 1 (PD-1/PD-L1) pathway has emerged as a treatment approach that provides a durable clinical response [1]. Anti-PD-1 therapy aims not only to improve the functions of immune cells but also to normalize the immune system, and thus exert its anticancer activity [2]. Currently, only ∼20% of patients achieve an objective response to immune checkpoint inhibitors (ICIs), while the others do not respond well or develop treatment resistance. Most ICIs are administered over the long term and come with the risk of significant toxicities. Additionally, patients’ responses to therapy can vary widely. Therefore, developing reliable predictive biomarkers is crucial for guiding individualized immunotherapy treatment [3, 4].

The *PDCD1* gene encodes human PD-1 (CD279) and belongs to the immunoglobulin gene superfamily. It was originally isolated by Ishida et al. [5] and was named for its involvement in apoptosis. It is a transmembrane glycoprotein that contains an extracellular IgV domain, a hydrophobic transmembrane domain, and a cytoplasmic tail structure domain [6]. An immunoreceptor inhibitory tyrosine-based switching motif (ITSM) located on the cytoplasmic tail appears to be necessary for the immunosuppressive function of PD-1 on T cells [7]. PD-L1 is a ligand of PD-1 and a type I glycoprotein which belongs to the protein B7 family. It contains IgV and IgC structural domains, a hydrophobic transmembrane domain, and a cytoplasmic tail structural domain [8].

PD-1 and PD-L1 are part of an essential signaling pathway that mediates immune tolerance in the tumor microenvironment. While PD-1 is a surface receptor of activated T and B cells, PD-L1 is expressed on tumor cells and antigen-presenting cells (APCs), but also in normal tissues, including epithelium, muscle, and placenta [9, 10]. When PD-1 and PD-L1 interact, phosphorylation of tyrosine residues in the PD-1 cytoplasmic region of ITSM occurs, recruiting the Src homology 2 domain-containing protein tyrosine phosphatase-2 (SHP-2). Subsequent phosphorylation of downstream proteins by the spleen tyrosine kinase (Syk) and phospholipid inositol 3-kinase (PI3K) inhibits T cell signaling and functions, such as proliferation, adhesion, cytokine production, and cytotoxicity [11, 12]. This process mediates the autoregulatory response and protects against local tissue damage during inflammation but can also result in tumor-specific T-cell exhaustion and apoptosis [13].

PD-L1 expression appears to mirror the dynamics between host immune surveillance and cancer immune escape. Thus, expression of PD-L1 in head and neck squamous cell cancer (HNSCC) increases along disease progression, being less frequent in premalignant lesions and most frequent in advanced disease [14]. Suppression of the PD-1/PD-L1 interaction can reset the immune system and enable it to attack tumor cells [15]. Several clinical studies have demonstrated the clinical activity of anti-PD-1/anti-PD-L1 agents in various tumors, including non-small cell lung cancer (NSCLC), melanoma, and Hodgkin lymphoma [16–18]. The overall findings suggest that the higher the expression of PD-L1 in cancer cells, the better the response to anti-PD1/anti-PD-L1 therapy. For example, in patients with NSCLCs, pembrolizumab and nivolumab achieved greater response in tumors with higher expression of PD-L1. Similarly, patients with Merkel cell carcinoma who responded to pembrolizumab had higher densities of PD-1+ and PD-L1+ cells when compared to non-responders [19]. Therefore, the presence of PD-L1 expression is considered a biomarker for anti-PD-1/PD-L1 treatment [20].

In 2011, the Food and Drug Administration (FDA) approved the first ICI for melanoma, ipilimumab (Bristol-Myers Squibb). In 2014, the FDA approved the first anti-PD-1 antibody, pembrolizumab (Merck), for metastatic melanoma. Other therapeutic monoclonal antibodies for NSCLC, urothelial carcinoma, and head and neck cancer followed soon after [21]. For the treatment of some cancers, such as NSCLC, the FDA requires a positive PD-L1 status before starting treatment with pembrolizumab [22]. Although several studies have reported a positive correlation between response to ICI, overall survival (OS), and positive PD-L1 expression, PD-L1-negative patients have also benefited from therapy. These results indicate that PD-L1 is not an independent and comprehensive biomarker. Its main shortcomings are the lack of a universal cutoff value for PD-L1 expression, the insufficient standardization of the PD-L1 assay and antibodies, and the spatial and dynamic heterogeneity of PD-L1 expression [23, 24].

In this review, we describe the different methods used to determine PD-1/PD-L1 expression, summarize the alternative methods used for PD-1/PD-L1 assessment, and present ways to increase the accuracy of PD-1/PD-L1 assessment. During the literature review, we analyzed the PMC, Embase, Cochrane Library, and Web of Science databases. Search terms included “PD-1”, “PD-L1”, “anti-PD-1/anti-PD-L1 immunotherapy”, “checkpoint inhibitors”, “PD-1 companion test”, “PD-1 complementary test”, “PD-1 digital assessment”, “PD-1 machine learning”, “anti-PD-1/anti-PD-L1 response”, and “anti-PD-1/anti-PD-L1 prediction”. The analysis was extended by searching the clinicaltrials.gov and fda.gov websites.

## Immunotherapy indications linked to PD-L1 expression and controversies

The immunohistochemical (IHC) PD-L1 staining of tumor tissue is used to predict response to therapy---in some indications patients’ PD-L1 status must be determined before the treatment can be started. The rules for categorizing test results as “positive” vary between different types of malignancies and relating to the quantitative threshold of stained objects and the scoring method used (Table 1). The evaluation of immunohistochemical staining is performed by humans---visually by a pathologist. It is therefore subject to certain variability. This problem had to be considered when developing methods for evaluating staining. Predictive tests used in practice must be carefully balanced and take into account two issues that do not necessarily coincide: prediction of response and a reasonably low level of complications that ensures high interobserver (and intraobserver) agreement. 

**Table 1:** PD-L1 testing and immune checkpoint inhibitor therapies (complementary or companion diagnostics)

**Sample type**	**PD-L1 testing**	**Expression cutoff value**	**Drug under consideration**
Cervical cancer	DAKO (22C3) companion diagnostic test*	CPS ≥ 1	Pembrolizumab
Esophageal squamous cell cancer	DAKO (22C3) companion diagnostic test*	CPS ≥ 10	Pembrolizumab
	DAKO (28-8) complementary test	TC ≥ 1%	Nivolumab
Gastric or gastroesophageal junction adenocarcinoma	DAKO (22C3) companion diagnostic test	CPS ≥ 1	Pembrolizumab
	DAKO (28-8) companion diagnostic test	TC ≥ 1%	Nivolumab
Melanoma	DAKO (28-8) complementary test	TC ≥ 1%	Nivolumab
NSCLC	DAKO (22C3) companion diagnostic test*	TPS ≥ 1%	Pembrolizumab
	DAKO (22C3) companion diagnostic test*	TPS ≥ 50%	Cemiplimab
	VENTANA PD-L1 (SP142) assay*	TC ≥ 50% or IC ≥ 10%	Atezolizumab
	DAKO (28-8) companion diagnostic test^1^*	TC ≥ 1%	Nivolumab + ipilimumab
	VENTANA PD-L1 (SP263) assay	TC ≥ 1%	Durvalumab
	VENTANA PD-L1 (SP263) assay	TC ≥ 50% (first line), TC ≥ 1% (second line)	Pembrolizumab
	VENTANA PD-L1 (SP263) assay	TC ≥1%, ≥5% and ≥10% (second line)	Nivolumab
	VENTANA PD-L1 (SP263) assay*	≥50% TC	Atezolizumab
	VENTANA PD-L1 (SP263) assay	TC ≥50%	Cemiplimab
SCCHN	DAKO (22C3) companion diagnostic test*	CPS ≥ 1	Pembrolizumab
	DAKO (28-8) complementary test	TC ≥ 1%	Nivolumab
Triple-negative breast cancer	DAKO (22C3) companion diagnostic test*	CPS ≥ 10	Pembrolizumab
Urothelial carcinoma	DAKO (22C3) companion diagnostic test	CPS ≥ 10	Pembrolizumab
	VENTANA PD-L1 (SP142) assay*	IC ≥ 5%	Atezolizumab
	DAKO (28-8) complementary test	TC ≥ 1%	Nivolumab

*FDA-approved Companion Diagnostic Devices, 22 Jun 2023 [173]; ^1^ only for NSCLC. **DAKO (22C3) Companion Diagnostic Test:** Tumor proportion score (TPS) is the percentage of viable tumor cells showing partial or complete membrane staining at any intensity. Combined positive score (CPS) is the number of PD-L1 staining cells (tumor cells, lymphocytes, and macrophages) divided by the total number of viable tumor cells multiplied by 100. **VENTANA PD-L1 (SP142) Assay:** IC is the percentage of tumor area covered by tumor-infiltrating immune cells. TC is the percentage of PD-L1-stained tumor cells. **DAKO (28-8) Complimentary Test:** PD-L1 protein expression is defined as the percentage of tumor cells exhibiting partial or complete membrane staining at any intensity. **VENTANA PD-L1 (SP263) Assay:** TC is the percentage of PD-L1 stained tumor cells. The percentage of tumor cells determines PD-L1 status with any membrane staining above the background or by the percentage of tumor-associated immune cells with staining (IC+) at any intensity above the background. The percent of tumor area occupied by any tumor-associated immune cells (immune cells present [ICP]) is used to determine IC+, which is the percent area of ICP exhibiting PD-L1 positive immune cell staining. PD-L1: Programmed death-ligand 1; NSCLC: Non-small cell lung cancer; SCCHN: Squamous cell carcinoma of head and neck; FDA: Food and Drug Administration; NSCLC: Non-small cell lung cancer.

There are four FDA-approved PD-L1 IHC staining protocols, consisting of four PD-L1 antibodies (SP142, 22C3, 28-8, and SP263) on two different IHC platforms (Agilent and Ventana Medical Systems Inc., Tucson, AZ, USA), each with its own scoring systems. There are also many FDA-approved assays for qualitative immunohistochemical assessment of PD-L1 protein [25]. Standardization and validation of these tests require pre-made test kits with reagents that run on company-specific staining platforms whose efficacy for the approved PD-1 and PD-L1 immunotherapeutics has been evaluated in clinical trials [26]. These tests can be divided into complementary (or co-diagnostic) assays and companion diagnostics. According to the FDA definition, complementary diagnostics may be related to a particular drug and provide more details about its potential use, but are not part of the approved indications. In contrast, companion diagnostics refer to a specific drug within its approval label to ensure its proper use [4, 27]. Diagnostic tests are complicated because performing each test requires the use of a different platform and its own antibody detecting system.

Currently, three types of scoring approaches are in use. The most straightforward—“tumor proportion score” (TPS) or “tumor cells” (TCs) parameter (the name varies depending on the manufacturer of the assay)—is defined as the percentage of PD-L1 positive tumor cells in relation to all viable tumor cells in a sample. The remaining two methods: “combined positive score” (CPS) and “immune cells” (ICs), introduce some intricacies. CPS adds another parameter to the TPS equation—immune cells. The score is then defined as the total number of viable tumor cells and immune cells in the tumor bed divided by the number of tumor cells only; the result is then multiplied by 100 to get an absolute number (with a maximum value of 100). The parameter IC is a percentage of the tumor surface area with a presence of PD-L1-positive immune cells relative to all tumor surface area. This method of assessment appears to be the least straightforward in practice. It is also inherently prone to arbitrary decisions regarding the border between an area occupied by inflammatory cells and the rest that is free of them. The IC assessment leads to low intraobserver agreement in some scenarios, and its applications are limited [28].

The arbitrariness in the evaluation of the staining score is not exclusive to the “IC” analysis. In practice, for example, it is virtually impossible to count tumor and/or inflammatory cells individually and calculate an accurate score because the cells of interest are usually present in the hundreds to tens of thousands. Routinely, then, the test result is an approximation—several types of malignancies have “about” a clear-cut impact on whether therapy will be used. Limitations inherent to the method can also have a significant impact on the evaluation. Known issues include artifactual (non-valid) edge staining and “perceptible and convincing membrane staining,” listed as valid in the evaluation manuals [29]. 

**Figure 1.**
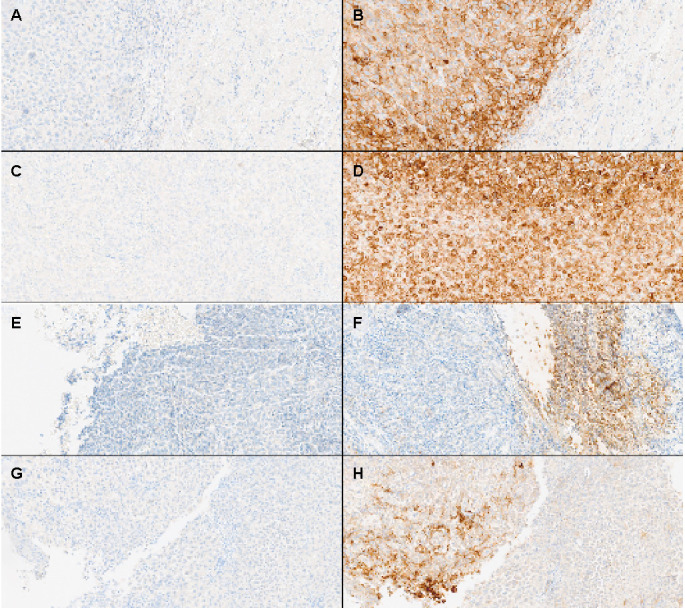
**Immunohistochemical patterns of PD-L1 expression stained by the SP263.** (A) Negative PD-L1 expression in head and neck cancer; (B) Positive PD-L1 expression in head and neck cancer; (C) Negative PD-L1 expression in lung cancer; (D) Positive PD-L1 expression in lung cancer; (E) Negative PD-L1 expression in triple-negative breast cancer; (F) Positive PD-L1 expression in triple-negative breast cancer; (G) Negative PD-L1 expression in gastric cancer; (H) Positive PD-L1 expression in gastric cancer. PD-L1: Programmed cell death ligand 1.

PD-L1 staining in tumor tissue may be heterogeneous, raising concerns about sampling reliability. A patient may have two different results of the PD-L1 staining test—positive or negative. For the administration of drugs, such as atezolizumab and pembrolizumab, a positive IHC test is required in some indications. For example, administration of pembrolizumab as a single agent for first-line treatment of patients with metastatic or unresectable recurrent HNSCC requires a CPS ≥ 1 [30–33]. Small specimens with a limited number of cells exacerbate this problem because they tend to show decreased staining intensity compared with resected material [34, 35]. Another issue regarding the IHC PD-L1 assessment is the variety of assays using specific antibody clones for FDA-approved drugs (Table 1). A comparative study has shown that assays are not always interchangeable, with clones 22C3, 28-8, and SP263 giving similar results, while SP142 is less intense and 73-10 is more intense in staining [28]. Parra et al. [26] found that Dako 22C3, Dako 28-8, and Ventana SP263 can be used interchangeably but not with the Ventana SP142 assay, which detected significantly fewer NSCLC cells. In HNSCC, the Ventana SP263 assay showed a higher rate of positive results than other assays, but the SP142 achieved the highest sensitivity (92%) and specificity (100%) in determining positive CPS scores. While these assays have a relatively high risk of false positive results, several studies are ongoing to determine whether certain IHC assays can accurately distinguish between PD-L1 negative and positive cells (Figure 1) [36, 37]. 

Future findings will play an essential role in the introduction of PD-1/PD-L1 targeted therapies and treatment of patients. 

## Solutions to enhance the viability of PD-L1 assessment prior to anti-PD-1/PD-L1 therapy

**Figure 2.**
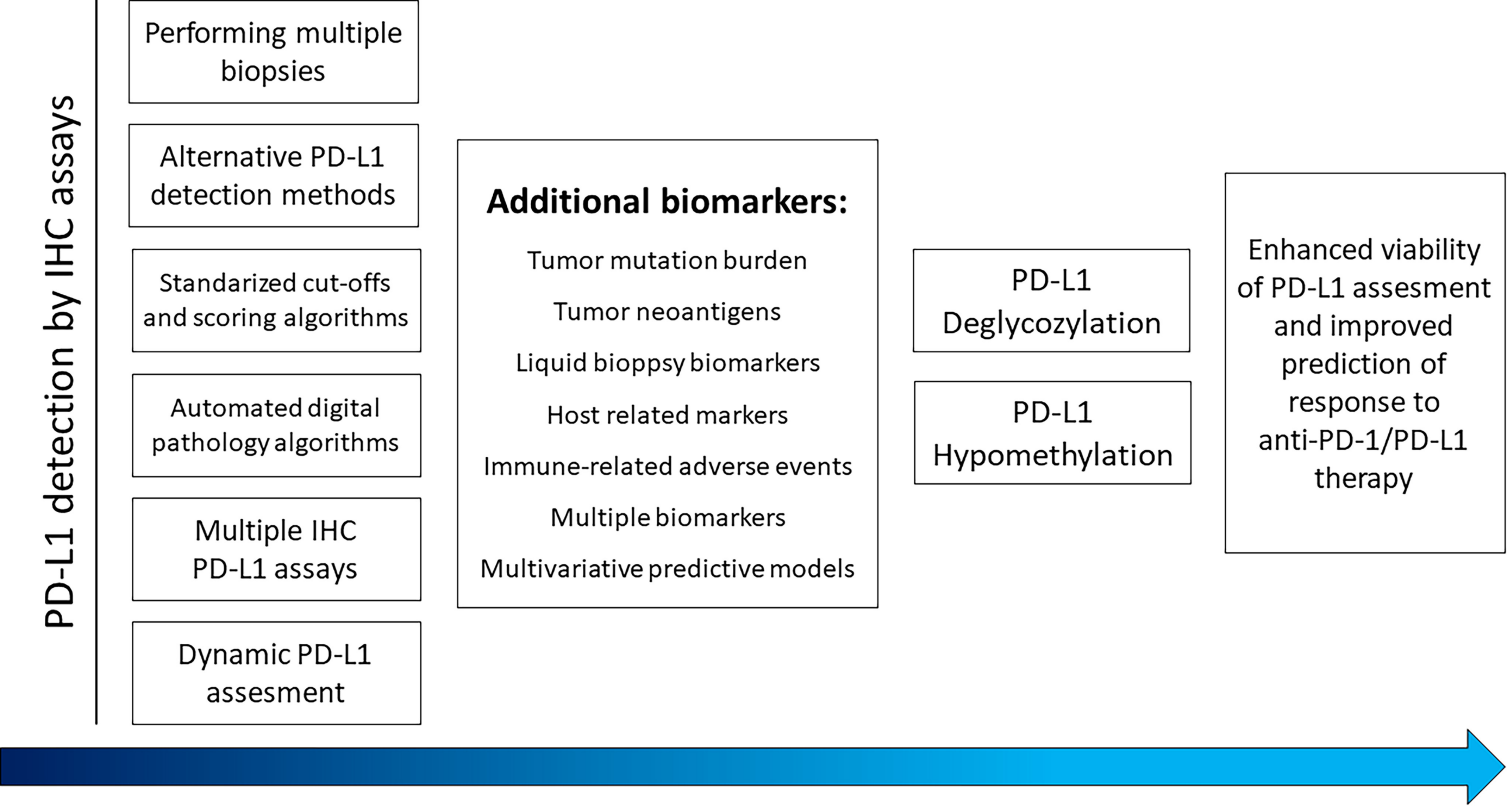
**The route to improving the discrimination between responders and non-responders before anti-PD-1/PD-L1 therapy.** IHC: Immunohistochemical; PD-1: Programmed cell death-1; PD-L1: Programmed cell death ligand 1.

To date, the population that might benefit from ICI therapy has not been optimally defined. Nevertheless, several factors predictive of response to PD-1/PD-L1 therapy have been identified, such as PD-1/PD-L1 expression, gut microbiome, antigen recognition, mismatch repair (MMR), microsatellite instability (MSI), tumor-infiltrating immune cells, and tumor mutation burden (TMB) [38]. To better exploit the therapeutic potential of PD-1/PD-L1 blockade, there is a need to identify predictive biomarkers of response to therapy, develop novel therapeutic strategies, and improve therapeutic strategies in combination with other agents. Novel negative predictive markers may potentially reduce the number of patients who do not respond to therapy. There is a great need for basic tumor immunology research and innovative clinical trials to fully unleash the potential of ICI combinations for the benefit of patients [39, 40]. In the next part, we will describe the approaches that improve the predictive power of PD-L1 assessment (Figure 2). 

### Assessment of the PD-L1 status

#### Performing multiple biopsies

The known heterogeneity of PD-L1 expression raises the question of how to ensure that the examined biopsy samples reflect the PD-L1 expression of the entire tumor. In their recent meta-analysis, Wang et al. showed no significant difference in the detection rate of PD-L1 at a 1% cutoff between biopsy and surgical resection specimens. However, there was a significant difference between the two groups when the cutoff was 50% (*P* < 0.01). The detection rate of PD-L1 in small biopsies using the SP142 antibody was lower than in surgical specimens, compared to using other antibodies for both 1% and 50% cutoffs (*P* < 0.01) [41]. Moreover, HNSCC biopsy specimens underestimated the prevalence of PD-L1, and their concordance with resection specimen results reached κ ═ 0.175 for 22C3 and κ ═ 0.266 for SP142 [42]. Because multiple tumors exhibit high intratumoral heterogeneity, diagnosis may require different cutoff values determined for each subtype of cancer; therefore, establishing a common PD-L1 cutoff value may be impossible [43, 44]. In addition, up to 35% of small (<2 mm) biopsies may be misclassified as either false negative or false positive compared to larger biopsies (>2 mm). It decreases to 10% when a threshold of 5 mm is applied, highlighting the need for adequate sampling for accurate PD-L1 evaluation [45]. Such analysis should take into account the differences between the primary tumor site and the metastasis [46, 47]. Munari et al. showed that at a 20% cutoff value for core biopsy specimens, fewer than three core biopsies are required to accurately identify cases of NSCLC (sensitivity >90%, AUT > 0.9). Three or four core biopsies were required to achieve high concordance at the 1% and 50% cutoffs. Thus, more defined sampling criteria could be helpful to more accurately determine the PD-L1 status of a given tumor [48–50]. Nevertheless, pathology studies comparing biopsies with resection specimens are not sufficient to provide a rationale for introducing additional biopsies into clinical practice. Interventions that affect the prevalence of positive and negative results require clinical validation, whereas the decision to perform additional biopsies should always consider the risks and benefits to patients. 

#### Alternative methods to detect PD-L1

IHC assays are not the only way to determine PD-L1 status. Quantitative immunofluorescence (QIF) provides sensitive and objective measurement of targets in user-defined tissue compartments. Briefly, the QIF score of PD-L1 signal for each antibody in tumor and stroma is calculated by dividing the target PD-L1 pixel intensities in the tumor and stroma compartments. McLaughlin et al. used QIF to show that PD-L1 expression assessed by E1L3N and SP142 was heterogeneous. Values for each tumor differed significantly by non-parametric paired test (*P* < 0.001). Over 25% of patients positive with one antibody were negative with the other. Expression of PD-L1 using both E1L3N and SP142 correlated with high tumor-infiltrating lymphocytes (*P* ═ 0.007 and *P* ═ 0.021, respectively) [51]. 

The CLOVER study estimated the compatibility between three IHC PD-L1 assays (22C3, SP142, and SP263) and the Taqman reverse transcription PCR (RT-PCR) test in NSCLC [52] The authors found high concordance between the TC scores of all three assays and weaker concordance between the IC scores. The correlations between the PCR result and the result of each IHC assay were low; however, if the PCR test was negative, there was also a high probability (92%–99%) that all IHC tests would also be negative. These results were recently confirmed by Venina et al. [53] and suggest that PCR-based analysis of PD-L1 expression is not suitable for detecting PD-L1-positive tumors. Although PCR can be used to identify PD-L1-negative tumors, it is not equivalent to IHC [52, 53]. 

Importantly, the above studies only assessed the concordance between QIF, RT-PCT, and IHC, not the accuracy of QIF and RT-PCR in predicting patient response to anti-PD-1/anti-PD-L1 therapy. These methods require controlled trials that would confirm their clinical validity compared with IHC.

Amplification of chromosome 9p24.1, which contains PD-L1, PD-L2, and Janus kinase 2 (JAK2), is commonly found in Hodgkin’s lymphoma, triple-negative breast cancer (TNBC), and NSCLC [54–56]. *PD-L1* and *PD-L2* gene copy number can be assessed by fluorescence in situ hybridization (FISH) or next-generation sequencing (NGS) [56]. In TNBC, the distribution of amplification of chromosome 9p24.1 targeting PD-L1, PD-L2, and JAK2 (PDJ amplicon) per cell varied significantly between biopsies. The mean copy number increased up to 26 copies in cells with PDJ amplification. Increased stromal tumor-infiltrating lymphocytes were detected in TNBCs with PDJ amplicons targeting PD-L1 and JAK2, suggesting that these cases may respond to ICI therapy [57]. Therefore, the increase in *PD-L1* gene copy number detected by FISH may be an alternative biomarker for predicting response to anti-PD-1/PD-L1 therapy [56]. 

Multiplex immunofluorescence (mIF) is based on tyramide signal amplification and multispectral imaging. It detects multiple markers in a single tissue section without affecting tissue architecture [58]. In a recent meta-analysis, multiplex IHC/IF appeared to have higher diagnostic accuracy than PD-L1 IHC alone [59]. PD-L1 mIF scores appear to correlate with the SP142 IHC assay and can be further improved by digital image analysis [60, 61].

Soluble PD-1, PD-L1, and PD-L2 in plasma can be measured by enzyme-linked immunosorbent assay (ELISA) [62]. Intra-assay imprecision measurements showed that the coefficient of variation did not exceed 10% for all three assays, while other analyses estimated good dilution linearity and selectivity of this method. ELISA can allow quantification of the dynamic expression of PD-L1, and a high sPD-L1 level predicts worse OS lung cancer patients treated with ICIs [63, 64]. sPD-L1 levels were also higher in renal cell carcinoma and melanoma patients with objective response than in patients with progressive disease [65]. Nevertheless, there are a few studies regarding using ELISE to predict patient response to ICI and compare its concordance with other diagnostic tools. 

### Assessment of PD-L1 expression dynamics

Surgery, chemotherapy, and immunotherapy affect the expression of PD-L1, which may increase when tumor volume decreases [66, 67]. Such heterogeneous expression of PD-L1 limits the applicability of IHC as a predictor of treatment outcome [68]. Real-time quantitative analysis of PD-L1 expression and dynamic mapping with radioisotope labeling can reduce the diagnostic uncertainty that arose due to PD-L1 level changes during treatment [69, 70]. Positron emission tomography and single-photon emission computed tomography (SPECT) can enable dynamic mapping of PD-L1. At the same time, the characteristics of SPECT/CT images using ^99m^Tc-NM-01-labeled anti-PD-L1 single-domain antibodies correlate with the results of PD-L1 IHC in NSCLC patients [71]. Diagnostic accuracy may also benefit from continuous sampling at multiple time points. However, the application of the above-mentioned approach might be quite complicated in daily practice [72].

### Evaluating PD-1/PD-L1 proximity

Recently, Nunes-Xavier et al. [73] reported that even patients classified as PD-L1-negative benefit clinically from ICI therapy, calling into question the validity of IHC in determining patients’ PD-L1 status. It appears that ligand expression is not sufficient to predict treatment response. Since PD-1 and PD-L1 mediate immunosuppression, assessing their proximity allows for obtaining additional information regarding their interactions [72]. 

iFRET is an imaging test that quantifies readouts of immune checkpoint interactions between cells and measures the distance between PD-L1/PD-1 on TCs and ICs [74]. Immune-FRET uses cell–cell amplified Föster resonance energy transfer detected by fluorescence lifetime imaging microscopy. First, PD-1 and PD-L1 are identified by their respective antibodies, and then the primary antibodies are stained with Fab conjugated to a donor chromophore: ATTO488 for PD-1 and ALEXA594 for PD-L1 [75]. When the distance is smaller than 1–10 nm, the fluorescence change is labeled as positive. The interaction between PD-1 and PD-L1 has also been detected in clear cell renal cell carcinoma, and their degree correlated with the prognosis of melanoma and NSCLC patients [72, 74]. 

mIF accurately quantifies the percentage of PD-L1 expression in NSCLC based on PD-1/PD-L1 interactions [76]. While six-plex mIF offers a high degree of control, simultaneous analysis of multiple markers, and evaluation at the single cell level, the reproducibility and technical requirements for single IHC assays appear to be more valuable in clinical practice [72]. 

### Automated digital pathology algorithms

Koelzer et al. [77] used HALOTMIA software (IndicaLabs, Albuquerque, NM, USA) to develop a PD-L1 expression scoring algorithm using an approach called “Random Forest” for melanoma. This trained algorithm recognized the stained and unstained membrane PD-L1 in tumor cells, while immune cells were successfully excluded and produced percentage of tumor cells. In the total cohort of 69 cutaneous melanomas, two independent pathologists found significant concordance between the automated PD-L1 analysis and the percentage tumor cell score. The protocol used for PD-L1 image analysis also showed excellent reproducibility [77]. 

The Aitrox AI Model assessed the expression of PD-L1 by categorizing TPS scores into negative (<1%), low expression (1%–49%), and high expression (TPS ≥ 50%) in NSCLC [78]. The authors compared TPS scoring results between AIrox AI, five experienced pathologists, and six inexperienced pathologists on whole-slide images (WSIs) to The Gold Standard for TPS from three experienced pathologists. Despite 5.09% of cases with large PD-L1 fluctuations, the Aitrox AI Model performed better than inexperienced pathologists and was comparable to experienced pathologists in samples with negative and low TPS. On the other hand, its performance was unsatisfactory in high TPS groups, especially when images contained large areas of false-positive cells. The predictions of Aitrox were similar to those of experienced pathologists (rs ═ 0.87), while its accuracy compared to pathologists varied depending on the TPS classification (85.29% vs 72.23%, low TPS and high TPS, respectively), indicating its potential use in assisted PD-L1 scoring [78].

Kim et al. [79] used the Aperio IHC membrane artificial intelligence (AI) algorithm (Leica Biosystems, Wetzlar) to compare pathologists’ interpretations and digital image analysis in gastric cancer. A total of 29 cases of gastric cancer were analyzed using PD-L1 22C3 PharmDX (Dako) immunohistochemistry slides, and the slides were interpreted by independent pathologists and digital image analysis. The concordance rate of these interpretations reached 84.6%, and the remaining findings did not differ significantly. In addition, a fully automated artificial algorithm for PD-L1 IHC staining was developed to analyze NSCLC needle biopsies. The algorithm used a novel machine learning approach called “generative adversarial networks.” In the study, automatic algorithm and manual scoring by a pathologist demonstrated high concordance [80]. Those findings illustrate that AI and digital pathology should be considered as a supportive tool for the analysis of PD-L1 expression.

## Advances in predictive biomarkers

### PD-L1 deglycosylation and hypomethylation

Glycosylation of PD-L1 prevents its degradation by the GSK3β-mediated 26S proteasome pathway and maintains its stability [81]. However, high levels of glycosylated PD-L1 prevent surface antigen recognition by anti-PD-L1 antibodies used in IHC assays [72, 81, 82]. Recombinant glycosidase removed N-linked glycosylation in A549 and BT-549 cells, enhancing PD-L1 fluorescence and increasing the binding affinity of antibodies compared to control [81]. Similar results were observed in colon cancer, where deglycosylation increased the intensity of PD-L1 signal in samples with low PD-L1 and reduced false-negative PD-L1 status [83]. However, the impact and prevalence of possible false-positive results remain unknown. Wang et al. suggest that deglycosylation of samples in an IHC protocol can remove N-glycans from surface antigens on formalin-fixed paraffin-embedded (FFPE) samples and increase binding affinity. Briefly, after pretreatment of the FFPE slide, denaturation of glycoproteins should be performed. Then, the slide is deglycosylated with a PNGase F and stained for IHC. Such adjustment could improve the correlation between PD-L1 assessment and clinical response [82]. 

DNA methylation can also alter the activity of the target gene and lead to different responses to treatment [84]. For example, a CpG-based model of Lasso predicts the overall response rate to PD-1/PD-L1 therapy better than models based on tumor mutational burden (AUC 0.92 vs 0.77). PD-L1 hypermethylation makes the disease less responsive to anti-PD-L1 treatment and is associated with shorter OS in several cancer types [72, 85, 86]. A positive correlation was observed between PD-L1 promoter methylation and PD-L1 expression in gastric cancer [87]. Methylation of PD-L1 promoter DNA predicted poor prognosis in melanoma, and hypomethylation of PD-L1 was associated with a transcriptomic phenotype [88]. The combination of hypomethylating agents and ICIs could improve treatment efficacy [85]. High ALKBH5 (m6a demethylase) inhibits m6a modification in PD-L1 DNA, which increases sensitivity to anti-PD-L1 therapy. Therefore, simultaneous detection of mPD-L1 and ALKBH5 may also improve the assessment of response rates to ICI treatment [89].

### Tumor genome and neoantigen biomarkers

Selection of antigens produced by tumor-specific mutations may enhance the efficacy of tumor-specific immune responses and minimize immune tolerance [90]. As DNA accumulates, the probability of successfully presenting neoantigens increases, as does the number of candidate peptides [91]. TMB significantly correlates with response to ICIs in various tumors [92]. In a recent meta-analysis, the objective response rate (ORR) to PD-1/PD-L1 therapy increased with TMB in 27 cancers [93]. Consequently, the National Comprehensive Cancer Network (NCCN) has adopted TMB for patients receiving immunotherapy for NSCLC. The FDA approved TMB as a diagnostic biomarker for pembrolizumab in April 2020 [94]. 

Different cutoff values for TMB were used in the different studies. The lowest cutoff was for nivolumab plus ipilimumab (>10 mt/Mb in NSCLC), while the highest was for pembrolizumab (>23.1 mt/Mb in NSCLC) [95]. Ming argued that high TMB, defined by >10 mutations/Mb, is not predictive of response across different cancer types. After testing several possible cutoffs, the author reported that a TMB cutoff of 13 mutations/Mb may be universally optimal for predicting favorable outcomes [96]. Another study using a TMB cutoff of 20mt/Mb in 4064 NSCLC patients with the Foundation One platform containing 395 gene panels found that OS and disease control rates were significantly improved in TMB-H patients compared to TMB-L patients treated with anti-PD-1/anti-PD-L1 drugs [97]. TMB ≥175 mutations/exome was associated with increased ORR (31.4% for TMB ≥175 vs 9.5% for TMB<175), longer progression-free survival (PFS), and longer OS in solid cancers treated with pembrolizumab [98]. In a recent meta-analysis, high TMB compared with low TMB predicted favorable PFS, OS, and ORR in NSCLC. In patients with high TMB, immunotherapy was associated with an improved response rate compared with chemotherapy. However, in patients with low TMB, immunotherapy was associated with shorter PFS and lower ORR than chemotherapy. This suggests that while high TMB may predict treatment response, tumors with high TMB may be more sensitive to chemotherapy than to immunotherapy [99].

Epigenetic changes are associated with TMB, and NSCLCs with high TMB have more DNA methylation copy number variations. This suggests a potential benefit in predicting *HOX* gene methylation status and TMB. Furthermore, the integration of DNA methylation and TMA data in NSCLC showed that patients with high TMB had more methylated CpG sites (mCpGs) than patients with low TMB [100]. The correlation between TMB and CpG methylation appears to be due to spontaneous deamination of methylated cytosines [101, 102]. In another study, the EPIMMUNE signature, an epigenetic signature of 301 different mCpGs, correlated with longer OS and PFS after PD-1 blockade. Interestingly, the results were independent of PD-L1 expression and TMB. Among known genes, a regulatory region of the forkhead box P1 gene showed the greatest difference in CpG methylation between responders and non-responders [103]. These epigenetic correlations paved the way for epigenetic studies based on liquid biopsies, indicating the next direction of research. 

Genetic variations in DNA MMR cause MSI, which is associated with a specific type of tumor that has a high number of DNA-based repetitions in a microsatellite. MSI, like PD-L1 and TMB, is one of only three FDA-approved biomarkers of response to immune checkpoint blockade (ICB). The durable response of patients in several clinical trials led the FDA to approve pembrolizumab for the treatment of all advanced solid tumors with MMR deficiency (dMMR/MSI-H) [104]. dMMR also causes mutations in the DNA polymerase gene epsilon/delta 1 (POLE/POLD1), increasing neoantigen load (NAL) and mutations. Studies on POLE/POLD1 mutations in various cancers showed that these mutations have higher TMB and OS [105]. The GARNET study (NCT02715284) tested the efficacy of dostarlimab, a PD-1 inhibitor previously approved for dMMR recurrent/advanced endometrial cancer, in patients with advanced and recurrent solid tumors. As shown, high TMB and PDL1 levels were common in dMMR solid tumors regardless of tumor type and correlated with a higher ORR (55.6% for all cohorts) [106]. 

Many other alterations in the tumor genome affect the efficacy of immunotherapy. Mutations in IFN-γ, JAK1/2, and other genes affect their respective signaling pathways and often lead to resistance and poor response to ICI therapy [107]. For example, anaplastic lymphoma kinase (ALK) and epidermal growth factor receptor (EGFR) mutations are associated with decreased response rates to ICIs and lower TMB scores. Therefore, for those patients, treatment with ICIs is not recommended as first-line therapy but may be considered after the failure of tyrosine kinase inhibitors [108, 109]. However, in KRAS-mutated NSCLC, the percentage of TMB and expression of PD-L1 seems to depend on the KRAS polymorphism, and its clinical role requires further investigation [110]. 

Neoantigen load (NAL) is directly related to response to ICIs. Tumors often consist of a variety of cancer subclones with different mutations that determine their response to therapy [111, 112]. Less heterogeneous tumors with fewer subclones than their parental cells had increased immunogenicity and slower growth [113]. Clonal antigens may induce an effective immune response, but some authors suggest that there is a threshold of antigen diversity beyond which T cells cannot control heterogeneous tumors [112, 114]. A high neoantigen burden in tumors with low neoantigen heterogeneity within the tumor is associated with a good prognosis. In primary lung adenocarcinomas, patients with high clonal neoantigen burden have longer OS (*P* ═ 0.025) [115]. Tumors with a higher TMB appear to have a higher neoantigen load (NAL) and are more likely to benefit from immunotherapy [116, 117]. A higher mutation burden is thought to generate more tumor-specific neoantigens that overexpress immune checkpoint modulators, such as PD-1 and PD-L1 [118]. The immunogenicity of the neoantigen can be assessed by analyzing its differential agretopicity index (DAI), which is defined as the ratio of binding affinities to the major histocompatibility complex (MHC) of the mutant and wild-type peptide. A higher DAI corresponds to the increased binding affinity to the MHC and increased immunogenicity [119]. Mutant peptides have a higher affinity for the MHC and a higher mean DAI compared to a non-mutated variant, which is associated with longer OS [94, 120]. DAI outperforms TMB in predicting ICI treatment outcomes and survival [121]. 

### Liquid biopsy biomarkers

Liquid biopsy is a non-invasive method for collecting fluid samples. To date, it has been mainly used to detect circulating tumor cells (CTCs) and cell-free circulating tumor DNA (ctDNA) [72, 122]. Liquid biopsy detects PD-L1 on the surface of CTCs and can monitor its dynamics in circulation during therapy, including shifts in soluble PD-L1, exosomal PD-L1, and PD-L1 expression in CTCs [72, 123–125]. 

In NSCLC and melanoma patients treated with nivolumab and pembrolizumab, respectively, neutrophil-to-lymphocyte ratio (NLR) is associated with poorer tumor response [126, 127]. In melanoma patients, NLR strongly predicted poorer response in patients treated with ICI, and high CD14+, CD16+, and HLA-DRhi cell levels predicted response to anti-PD-1 therapy [128, 129]. Elevated IL-8 levels predicted a negative response to anti-PD-1 and anti-CTLA-4 in patients with metastatic melanoma and NSCLC [130]. 

Another thoroughly studied biomarker that can be assessed by liquid biopsy is CTCs. CTCs are commonly used to evaluate response to therapy, as they have shown higher sensitivity than imaging studies in some cases [131]. As a non-invasive method, CTC analysis can avoid the frequent radiation exposure associated with imaging studies [132]. They are a highly heterogeneous group that arise during the epithelial–mesenchymal transition and acquire a mesenchymal-like phenotype. These changes facilitate their escape from immune response and resistance to immunotherapy [133]. Persistence of PD-L1-positive CTCs after six months of treatment with nivolumab has been associated with disease progression in NSCLC patients [134]. 

On the other hand, the absence of PD-L1-positive CTCs or a decrease in the number of CTCs predicted a better prognosis and a sustained response to long-term immunotherapy [135, 136]. However, in some studies, no decrease in CTC was observed after immunotherapy, and CTC assessment was not included in RECIST guidelines [132, 137]. Currently, the large heterogeneity of CTC is considered one of the greatest obstacles to its widespread use in predicting patient outcomes. Nevertheless, recently developed technologies that allow genotyping of CTC may be the next step toward truly personalized therapy [138, 139]. 

### Circulating tumor DNA biomarkers

Genomic information about the tumor provided by ctDNA can be used to predict response to ICIs; however, the sensitivity and specificity of this method still need to be improved. In patients with metastatic melanoma, ctDNA correlated with increased TMB. At the same time, undetectable ctDNA before treatment correlated with longer OS and PFS, and high ctDNA at baseline was a predictor of poorer prognosis [140–143]. Persistent elevation of ctDNA during ICIs treatment corresponded to poorer response to immunotherapy, worse OS, and worse prognosis. It can also assess pseudoprogression after ICIs therapy [142].

### Host-related markers

Host characteristics also influence the response to immunotherapy. The meta-analysis by Conforti et al. [144] showed that gender was associated with a higher response rate to antitumor immunotherapies and men treated with ICIs had a lower hazard ratio than women (0.72 vs 0.86; *P* ═ 0.0019). In another meta-analysis, male patients treated for NSCLC and melanoma had longer PFS and OS compared with female patients [145]. In contrast, gender did not affect PFS, OS, and IRR in clinical practice in the study by Choi et al. [146]. 

In several studies, an increased intestinal microbiota appeared to increase the percentage of patients responding to ICIs [94]. A more numerous “good” microbiota was found in melanoma patients who responded to treatment [147]. In a meta-analysis on melanoma treated with anti-PD-1 drugs, microbiota composition was associated with outcomes one year after initial treatment. *Actinobacteria phylum, Lachnospiraceae, and Ruminococcaceae* family of *Firmicutes* were associated with favorable prognosis. Gram-negative bacteria, on the other hand, were associated with unfavorable outcomes. Microbiota signatures enriched in *Lachnospiraceae spp.* and *Streptococcaceae* were also associated with favorable and unfavorable clinical outcomes, respectively [148]. An analysis of patients treated with ICIs showed that HLA-I was associated with prolonged survival due to the increased number of tumor antigens [149].

With long-term administration, ICIs can enhance the immune response and cause immune-related adverse events (irAEs) [150]. Although irAEs appear to be idiosyncratic, they are neither a laboratory test nor a biomarker, but rather a manifestation of immune system activity. As autoimmune conditions that occur after ICI administration, irAEs can affect any organ and differ in their natural history from autoimmune diseases that arise de novo [151]. Therefore, these toxicities present multiple challenges in clinical practice and require a steep learning curve to diagnose and treatment. irAEs that occur early after treatment initiation are associated with favorable PFS and ORR in NSCLC patients treated with nivolumab [150]. Patients with gastric adenocarcinoma with irAEs treated with anti-PD-1 antibodies had a longer OS (176 days vs 94 days, *P* ═ 0.001) than patients without irAEs, and the occurrence of irAEs was an independent favorable prognostic factor in this group [152]. 

Different immunotherapeutic responses are associated with different irAEs. For example, endocrine irAEs are associated with better prognosis and OS in melanoma patients, while thyroid dysfunction relative to anti-PD-1 treatment in NSCLC patients predicts longer PFS and OS [153, 154]. In the meta-analysis by Wang et al. [155] irAEs were associated with higher ORR and OR in lung cancer patients who underwent ICI. The prolonged OS remained significant for dermatologic, endocrine, and gastrointestinal irAEs, but not for hepatobiliary, pulmonary, and high-grade (≥3) irAEs. Zhang et al. [156] reported similar correlations in NSCLC patients.

### Predictive biomarkers by ICI type

Specific biomarkers can predict clinical response to various ICIs. High expression of anti-CTLA-4 resistance-associated MAGE-A (CRMA), an eight-gene cluster, predicts poor response to anti-CTLA-4 therapy but not to anti-PD-1 therapy. Therefore, CRMA can be used to identify patients who do not respond to anti-CTLA-4 therapy and may require anti-PD-1 agents instead [157]. MHC molecules have different sensitivity and efficacy to CTLA-4- and PD-1-targeting antibodies. Loss of MHC-I expression in melanoma cells leads to resistance to anti-CTLA-4 but not anti-PD-1 therapy. On the other hand, expression of MHC-II, which correlates with IFN-γ, has been shown to predict melanoma response to anti-PD-1 but not to anti-CTLA-4 therapy. It appears that MHC-I expression elicits a response against CTLA-4 melanoma, whereas an anti-PD-1 response requires a pre-existing IFN-γ-mediated immune activation [158].

### Combination of multiple biomarkers

Single biomarkers often lack sensitivity and specificity to reliably predict response to ICIs. A combination of multiple factors, such as TMB, PD-L1, and CD8+TIL, is required to perform a more accurate assessment. PD-L1 expression and high TMB are associated with high benefit rate, positive predictive value of ORR, and longer PFS in NSCLC patients [159, 160]. Similarly, Yu et al. showed that a combination of CD8+TIL, PD-L1, and high TMB improved PFS and OS prediction compared with a single biomarker [159]. The combination of TMB ≥10 mutations/Mb, PD-L1 > 50%, and NLR <5 appears to improve the prediction of ICB outcome in NSCLC patients compared to TMB ≥ 9.24 mutations/Mb alone (AUC 0.62 vs 0.74). The combination of high TMB, positive PD-L1, and low NLR correlated with longer OS (*P* ═ 0.038) but not with time to progression [161]. 

The IMAGiC model predicts response to ICI in patients with advanced gastric cancer based on the expression signature of four genes (ubiquitin C-terminal hydrolase L1 [UCHL1], tyrosine kinase 2 [TYK2], protein kinase D1 [PRKD1], and armadillo repeat-containing X-Linked 1 [ARMCX1]). The cutoff value was set at −0.18, and patients with values below −0.18 were classified as IMAGiC responders. Responders achieved a higher complete response to partial response ratio and longer median PFS than non-responders (70.6 vs 21.4% and 20.8 months vs 6.7 months, respectively) [162]. 

The ICB treatment signature (ITS) score consists of selected genes associated with high CTL levels and TMB but excludes genes related to immunosuppression. NSCLC patients with a high ITS score had longer PFS and higher ORR (53.8% vs 7.1%) after ICB treatment than patients with a low ITS score. A higher ITS score was an independent prognostic factor for favorable prognosis (HR ═ 0.097, *P* ═ 0.02) [163]. 

Goodman et al. employed the Patient Harmonic-mean Best Rank (PHBR) and TMB to explain the interaction between TMB and MHC-I and predict the response to ICB. A low PHBR represents strong neoantigen presentation, and a high PHBR represents poor neoantigen presentation. The median PFS for PHBR score < 0.5 vs ≥ 0.5 was 5.1 vs 4.4 months (*P* ═ 0.04). Using a TMB cutoff ≥ 10 mutations/Mb, patients with TMB high/PHBR high had a lower response rate (43% vs 78%) and a shorter median PFS (5.8 vs 26.8 months) than patients with TMB high/PHBR low. These results suggest that insufficient presentation of neoantigens may contribute to poor response to ICB [164].

## Machine deep learning and artificial intelligence in developing multivariate predictive models

In recent years, the role of AI and machine learning in cancer research has increased, offering pathologists help in increasing diagnostic accuracy [165–168]. Several studies describe automated PD-L1 scoring using AI [169, 170]. 

Chen et al. are developing a deep-learning model to predict IHC phenotype directly from WSIs to facilitate lung cancer subtyping. In the validation databases, the area under the curve (AUC) and overall diagnostic accuracy of the algorithm averaged 0.906 and 0.941 for surgical resection specimens and 0.888 and 0.887 for biopsies, respectively. Overall subtyping performance was similar to that of a general pathologist. Furthermore, when the authors tried to determine whether they could use small biopsy specimens to train the system to predict the expression status of the proteins ALK, PD-1, and PD-L1, the AUCs reached 0.917, 0.576, and 0.525 for ALK, PD-1, and PD-L1, respectively [166]. Therefore, other algorithms may be more suitable to accurately detect PD-L1 status.

Shamai et al. used annotation software to develop a system that predicts PD-L1 status from H&E-stained histopathologic images of breast cancer. The system was validated in two external datasets and showed consistently high performance, with an AUC of 0.91–0.93. Wang et al. used deep learning, radiomics—a method that extracts many features from medical images—and computed tomography-based models to non-invasively measure PD-L1 expression in NSCLC. The authors used 3D ResNet as a feature map extractor for deep learning and constructed a specialized classifier. The AI algorithm showed accurate prediction of PD-1 status and achieved an AUC >0.93 for all cutoffs tested [171]. 

Deep learning image analysis offers an alternative to IHC PD-L1 assessment. Considering the continuous progress in AI and the emergence of systems that identify cases prone to misinterpretation by pathologists, they may soon support decision making and ensure quality control in clinical practice [172]. 

## Conclusion

Anti-PD-1/PD-L1 therapy has significantly improved clinical outcomes in numerous malignancies. However, due to long-term drug administration, potentially serious adverse events, and unsatisfactory concordance between different PD-L1 assays, it remains a challenge to improve the viability of biomarkers that predict response to ICIs. Heterogeneity within the tumor prevents the establishment of a universal cutoff value for PD-L1 assessment, while biopsy specimens usually underestimate PD-L1 expression, indicating that diagnosis may require subtype-by-subtype analysis. Although performing multiple biopsies may increase diagnostic accuracy, such an intervention should be preceded by randomized-controlled trials. 

Alternative methods for detecting PD-L1 by QIF, RT-PCR, mIF, iFRET, or ELISE showed high agreement with the results of IHC assays, but their suitability for predicting patient response to ICIs remains to be determined in clinical practice. Preanalytical modification during sample preparation, such as PD-L1 glycosylation by PNGase F in formalin-fixed, paraffin-embedded samples, may increase the binding affinity of the anti-PD-L1 antibody and improve prediction of clinical response. A high neoantigen and TMB reflects high intratumoral heterogeneity and is associated with a good response to immunotherapy. However, their accuracy depends on the activity of the host immune response and may be inaccurate in case of insufficient antigen presentation. Recent reports suggest that methods, such as the Differential Agretopicity Index, may be better suited to predict patient response.

Analysis of PD-L1 expression on the surface of CTCs and genomic data from ctDNA provides a dynamic assessment of disease course. The disappearance of PD-L1-positive cells after therapy predicts a sustained response to long-term immunotherapy. Recent reports suggest that the combination of multiple biomarkers, either based on TMB, PD-L1 expression, NLR, or gene expression signature, has higher sensitivity and specificity in predicting clinical response than single biomarkers. Nevertheless, these models need to be tested in clinical trials.

Finally, the development of digital pathology and machine learning brought fresh air to the field of biomarker analysis. Algorithms based on whole-slide histopathological or computed tomography image analysis show high performance and can identify cases susceptible to misinterpretation by pathologists. Although their use as the sole method for predicting clinical response is questionable, they may soon support decision making and become a quality control measure.
